# On the Organization of Asylums for the Insane
*This essay was presented to the "Association of Medical Superintendents of American Institutions for the Insane," in June, 1850. It has been published in the "American Journal of Insanity."


**Published:** 1852-10-01

**Authors:** John M. Galt

**Affiliations:** PHYSICIAN TO THE EASTERN ASYLUM OF VIRGINIA, AT WILLIAMSBURG


### ON THE ORGANIZATION OF ASYLUMS FOR THE INSANE.*

BY JOHN M. GALT, M.D., PHYSICIAN TO THE EASTERN ASYLUM OF VIRGINIA, AT WILLIAMSBURG.

At the last meeting of the Association of Medical Superintendents of American Institutions for the Insane, the state of my health prevented a participation in the interesting proceedings of this body. Having been directed, according to an order passed at a previous session, to choose a subject on which to report, I did so, but was unable to write out my views fully, owing to the same reason just given above for my absence from the meeting referred to. Insomuch, however, as most of what I should have remarked would have necessarily been found elsewhere, and doubtless expressed in a better manner than any effort of mine could attain, it matters little that this duty was unfulfilled. But wishing to conform as closely as possible to aught assuming the shape of a promise, I content myself with now presenting a few observations concerning the Organization of Asylums for the Insane, instead of offering an elaborate article on the subject. I shall therefore simply touch upon three prominent points in this relation.

The first of these topics which we proceed to notice, is an arrangement suggested in connexion with the early history of a lunatic asylum: we think that when such an institution is contemplated, the medical superintendent thereof should be appointed before the building is put up, or even a plan of construction is adopted. And this, too, whatsoever be his particular functions with regard to the necessary buildings; in other words, for example, whether he be entrusted with the supervision of the whole undertaking, or have only the task of making suggestions as to the adoption of a suitable model. Not unfrequently we find that it is a practice with the trustees of new asylums to select as their medical superintendent, some gentleman who is already connected officially with an establishment of the kind. In such event, as regards an asylum designed to be erected, it is evident that you secure the aid resulting from the counsels of an individual directly and personally interested in the proposed institution, who has a thorough acquaintance with the architectural requirements for the management of the insane. Here you have, therefore, the combined assistance of self-interest, experience, and study. But even in cases where an ordinary physician is selected, you have under this plan, in the first place, an early attention on his part to the important subject of architectural provisions; and, secondly, almost as a matter of course, a devoted investigation into the general subject of insanity and its treatment. Hence, when the establishment is ready to be occupied by its future insane inmates, the superintendent is fully prepared to undertake its judicious supervision.

†Secondly, the government of an asylum as at present constituted, usually

* This essay was presented to the "Association of Medical Superintendents of American Institutions for the Insane," in June, 1850. It has been published in the "American Journal of Insanity."

† At their meeting, held in May, 1848, in the city of New York, the Association of Medical Superintendents of American Institutions for the Insane adopted the following preamble and resolution relative to the appointment of superintendents:—

Whereas, in the selection of Medical Superintendents to American Institutions for the Insane, it is important to choose men with the highest qualifications, both as respects professional acquirements and moral endowments, therefore

*Resolved*, That any attempt, in any part of this country, to select such officers through political bias, be deprecated by the association as a dangerous departure from that sound rule which should govern every appointing power, of seeking the best men, irrespective of every other consideration.

consists of a Board of Trustees, and a superintendent acting under their direction. I am of opinion, that all persons whatsoever, serving in the capacity of assistants to this officer, should be absolutely under his control as to dismissal from their situations; and that every such assistant should either be selected by the superintendent immediately, or from his nomination to the Board of Trustees; the superintendent being himself appointed by that body, and being liable to removal through their action. This last regulation I deem a sufficient check upon the superintendent, whether he be given the power of nomination or that of appointing. He is more accurately acquainted with the precise characteristics which are requisite in any subordinate than the Board of Trustees can possibly be; experience, observation, and self-interest teach him this, and on these grounds merely, it is far more likely that he will make a good choice than they. Then again, if an individual is found on trial not to possess the requisite qualifications, of which circumstance the superintendent alone is the proper judge, a new appointment can be effected without the exciting and prejudicial process of an examination into the matter on the part of a superior authority. Moreover, such bodies are unpaid, and to some extent irresponsible; and it is contrary to human nature to suppose, as an ordinary event, that they will take so lively an interest in the welfare of an establishment of the kind as is evinced by most superintendents; hence they would be more easily induced, through the persuasions of others, to give their votes for persons not exactly qualified for particular posts in an asylum. Whereas this is very different with a superintendent. For it is pretty certain that the success of an institution for the insane depends greatly upon the character of this officer's subordinate auxiliaries, and therefore it is to his direct advantage to choose those assistants that will faithfully and efficiently perform their several duties. There is, indeed, a sure guarantee of proper management here, in the fact that a failure of success is, in the chief officer, a failure in the mode of earning his livelihood and supporting his family; and the risk in connexion with incapable subordinates is of extreme importance to him. So far as the power of dismissal is concerned, it is manifest to all who have had charge of the insane, that there are officers and attendants whose deficiencies cannot be well explained in words, or fully demonstrated and pointed out for the decision of trustees, and yet an institution may suffer grievously from the presence of such individuals. Moreover, no general system can be fully carried out, unless each member of the official corps co-operates fully with him who has the responsibility alike of both the medical and the domestic arrangements of an asylum; and it is in vain to expect so desirable an union of effort, where subordinates look to a higher power than the superintendent.*

* "In the French establishments, the visiting physician is invested with supreme power over the medical and moral management, appoints the attendants and some of the subordinate officers."—I. Ray, M.D., Superintendent of the Butler Hospital for the Insane, at Providence, Rhode Island. Whilst speaking of the action of trustees in England, in a policy the reverse of that just mentioned, Dr. Ray observes: "In the exercise of the appointing power, a favour is dispensed and an obligation is incurred, that may redound in some way to the benefit of the obliging party. True, the helping of a servant to a place would seem to be a privilege hardly corresponding to the dignity of the class who exercise it, but this consideration is allowed to have but little influence, when a dependent may be placed beyond the want of future assistance, or a powerful friend obliged. At the very least, the simple abstract love of patronage is gratified, and that is something to those who may never have had the opportunity before."

"There is one (topic), however, to which I will call your attention, because it lies at the foundation of a successful, permanent organization, and is, in my opinion, the only safe basis upon which a lunatic hospital can be organized: which is, that the superintendent should be a physician, with the entire control of all departments of the institution, domestic as well as medical (of course under the direction of a board of trustees). The superintendent should have this control, because unity of action, arising

Comfortable accommodations, liberal diet, and warm clothing, now constitute established, settled means of treatment in insanity. These are forms of expenditure that *must* be incurred, and little difficulty is usually found in obtaining them. But if there be any progress in the treatment of this disease, if there be any measure radiant with future promise, it is discoverable in the agency of the influence over the minds of the insane, that results from the exertions of teachers and other additional companions appointed for their benefit. It is here, therefore, that the battle is to be fought for an increased outlay in our lunatic asylums. But certainly we should seek that medium in this regard, by which we can obtain the largest available force of such agents at the least expense. Here, too, the simple though important doctrine of political economy should always be held in due consideration, to the effect, that in every department of public business the people should be served by individuals fully qualified for the discharge of their official duties, and that an expenditure should be allowed, requisite for securing the services of such persons; but that any amount beyond this must be considered as wasteful extravagance. The more closely indeed we adhere to the rule thus enunciated, the greater will be the number of our assistants in so material a line of endeavour. Legislative bodies may rest assured, that never will the combined advantages of proper treatment and minimum expenditure be fully attained, until the superintendent has the control of his subordinates, for which I have contended above. Under the old system of organization, where the steward and matron, and a few other officers and attendants of very definite functions, formed the entire body of agents assisting the superintendent, the exact degree of compensation involved in obtaining the services of persons capable of filling a particular office, was more easily assignable and capable of recognition. But when, in addition, we have teachers and companions, and, in fine, a much greater variety of capabilities in demand, to procure an entire set of officials, all of the requisite character, becomes difficult, and especially so in conjunction with motives of economy. Owing to his practical experience, a superintendent alone can determine with accuracy the comparative facility in obtaining talent or natural ability suitable to each post in an asylum; so as measurably to graduate the salaries according to this scale. It is a simple matter for a subordinate officer, apparently faithful to his trust, to represent to a Board of Trustees the onerous nature of his duties, and, by such a course, to induce a useless increase of salary. But the superintendent is alone capable of judging properly, first, the fidelity and value from unity of views and sentiments, is the chief element of system. System cannot exist if the action comes from more than one source; and without system, there cannot be success. Upon him should responsibility rest, as under any arrangement in public estimation it will rest; he should have the entire control; his spirit, his plans, his system, should pervade the institution; from him all power should proceed, that consequently when he delegates to others the duties of the several departments, those duties should be performed in accordance with that system, however much the opinions of subordinate officers may differ from his. The more entire the control, the greater safety in delegating to others those subordinate duties; and this truth is well illustrated in the best-arranged and the best-managed hospital in this county (Worcester), where the superintendent (nominating the steward to the board and appointing all the officers) having the entire sway, derives the greatest assistance from, and reposes the utmost confidence in, his subordinate officers. They adopt his system and carry it out. There is no clashing of conflicting opinions; there can be none where one system pervades the whole. This principle is adopted in all the departments of associated efforts in society, and is nowhere more essential to successful results. The guards against the possible abuse of this concentration of power in one individual, are to be found in the frequent and rigid inspection by the trustees, of every department and room in the hospital, and the free access and invited scrutiny of an intelligent community. Under such an oversight, it is not possible, in this country, for any erroneous practices to be kept long concealed from the public eye."—*Dr. John S. Butler.*

of any particular assistant; and, secondly, the amount of pay which he should receive, from the comparative facility of filling the situation which he holds. Let the superintendent but have the power of appointment and dismissal, and he is perfectly aware when he should discharge an official who is dissatisfied, and when, on the contrary, he should recommend an increase of salary to one whose ability could not easily be found in another.*

The third point to which I would call the attention of the Association, is the question whether it is advisable to have a visiting or a consulting physician, instead of the American plan of dispensing with such an official.† I think this strict exclusion to be at least a doubtful policy. Now, where, as in some few asylums in this country, and in a large number on the other side of the Atlantic, the visiting has superior authority over the resident physician, no doubt, in adopting the plan, we should be establishing one inferior in merit to that in vogue amongst us—however well the former may have succeeded in particular institutions of the United States. But the arrangement which I would propose is, that the superintendent should have the nomination or appointment of a consulting physician, who would thus, like the other officers, be considered as an auxiliary subordinate. This officer might or might not be recompensed pecuniarily. Whilst in private practice, scarcely a person becomes dangerously ill, but that their friends view it as necessary to call in more than one physician, should not the same rule apply, if not to insanity as a disease, at least to formidable maladies, to which the insane are equally as liable as are those of sound mind? Were there any power given to such an officer which would conflict with the authority of the superintendent of an asylum, I should be clearly against so undesirable an arrangement; but under that which I propose, nothing of the kind is admissible. The officer in question is to be selected by the superintendent, and consulted by him when deemed necessary. If it be alleged that this arrangement endangers the growth of cabals and intrigues against the superintendent, the answer is simply, that physicians are found in all Boards of Trustees—in other words, occupying a position superior in point of fact to that of this officer; and yet these gentlemen are oftener of service to him than the reverse. Apart from the old adage as to the increased wisdom in numbers, an important advantage under the plan pursued, would be attained by giving satisfactory testimony to the friends of patients as to the care taken of their afflicted relatives; for they thus perceive that these unfortunates have not only the benefit arising from the enlarged experience of the superintendent of an asylum as to mental derangement, in which particular he almost necessarily excels other medical men, but also that on the occurrence of bodily disease, they would have attendance of a character not to be surpassed at home. A second advantage in this regard consists in the circumstance, that in many instances the diseases prevalent in the vicinity of an asylum would be known by a consulting physician, who, as a general rule, would be probably a physician in practice, and thus additional light might be constantly thrown on the physical diseases from time to time attacking the inmates of an institution in an endemic or epidemic form. Again, the false reports and rumours occasionally affecting the reputation of an asylum for the insane, could not receive a more useful contradiction than would come from the lips of a physician in active practice. In the third place, as to many difficulties, not only with regard to treatment, but as to general management also, an influential physician, by his counsel, and by his testimony out of doors, might often lighten the weighty load of responsibility to which every

* As regards the offices of steward and matron, I may remark, *par parenthèse*, that I should consider their abolishment a desirable innovation.

† "In such a case, we cannot doubt that the frequent visits of an intelligent physician in general practice may be, in a variety of ways, of the greatest advantage."—*Samuel Tuke.*

superintendent is subject; and suggestions of improvement would not unfrequently occur to such an officer, which might escape even the experienced mind of a superintendent, burdened as he must ever be by a multiplicity of cares and multiform duties.

Moreover, and lastly, by filling the office of consulting physician, instead of that of superintendent, lives valuable to the cause of the suffering insane might be prolonged for years, which, under the toils of a superintendency, would be quenched in darkness, after shedding for a short time a brief and transitory, though effulgent light. In this connexion, I trust it will not be deemed amiss to offer one humble tribute of admiration to the memory of the lamented Brigham—from the south, to add one more to the many voices which have uttered their praise of his exalted merits. If this eminent labourer in the field of benevolence, after establishing on a permanent basis the important charity over which he so ably presided, had then acted in its behalf, under a less confining class of duties—a situation which would but have given more scope for his sensible suggestions and his fearless reflections, we might still, perchance, have had the light of his intelligence amongst us. He might have been a blessing for years to the great institution in whose service he died, as the martyrs of old offered their corruptible bodies for an incorruptible faith. He might have been a blessing for years to the insane in the populous commonwealth which chose him from afar to watch over the infancy of its noble asylum; to the insane in every State of this extensive and expanding Union, in whose cause his wise words will ever be as a beacon and light to those who would strive for their benefit; to the mentally afflicted, in fine, everywhere: for his was a most liberal sympathy, and was displayed for the good of all in every land, whose minds are darkened. But alas! he has gone from us for ever! Ours is the loss—his the exceeding reward. Whilst on this earth, he contended for the truth against all opposition and under all circumstances. He is now gone to that mighty Being who is the source and essence of truth. His spirit has passed to the bosom of the Eternal One, where the toil-worn and weary have an everlasting rest.

### ON THE MEDICO-LEGAL QUESTION OF THE CONFINEMENT OF THE INSANE.

The subject on which I have been instructed to report, is somewhat peculiar, in the fact that it may be referred conjointly to two important professions—medicine and law. With regard to the considerations which appertain to the first half of the compound term, the indications for the confinement of persons labouring under insanity are manifest in a number of cases. Taking those which are clearly proved to belong to mental derangement, it is obvious, for example, that the medical treatment will be very uncertain, if the patient is allowed to go at large and to act according to his own fancy. In most instances, which at all approach the maniacal type, the individual then must be confined on his own account, for his own welfare. Here any scruples as to personal rights are necessarily to be waived, being dispelled by the advantages which accrue to the patient himself from placing him either in positive confinement, or under such a degree of control as will enable the physician to give suitable directions in the way of treatment, and further, to ensure the certainty of these directions being carried into effect. In this view, and under the present head, what is entitled moral treatment and the deductions in connexion with it, fall under the general division of medical treatment, as contradistinguished from the second question, or the considerations arising as to the legal necessity of restrictions on the insane. Upon the ground so stated, another principle for confining this class is found in the circumstance, that should the patient be left to indulge his peculiar morbid ideas and propensities unchecked, there is an increased intensity given to them; hence, one of the rules of moral management, to lessen the force of these, by exciting in the diseased mind new trains of healthy thoughts and emotions; but to effect this, presupposes the exercise of a due degree of control over the individual. If for these and other reasons which might be mentioned, it be both justifiable and judicious to confine a lunatic at home or elsewhere, so far as the benefit from medical supervision is concerned, the argument has the greater force when applied to isolation in an asylum, because here the means of effecting good results through the agency of treatment, are much more efficient and extensive than in general could be provided in any other situation. This conclusion has been so universal, that it scarcely seems necessary, either to enter into the comparative merits of treatment in asylums properly managed and constituted, and that pursued elsewhere, or, on the other hand, to discuss the essential difference as to various points between the two modes of action. Suffice it to state, that medical authorities in all civilized countries are agreed as to the superiority of asylums in this regard. And there is not a doubt on the score of humanity, that this greater efficacy altogether justifies the increased abridgment of liberty, which is sometimes the lot of the insane when thus situated. Whilst we consider, however, the lamentable condition of those confined in prisons, and also of some in confinement at home—whilst we view with feelings of compassion the utter misery attending the situation of a large number of these unfortunates in every land, we cannot but conclude that the natural liberty of a citizen is practically and in reality far more affected by a residence in localities like these, than when he dwells within the precincts of an asylum. In such an establishment, the mournful isolation of dark and loathsome dens, and the degradation of chains and stripes, are done away with entirely, and the hapless lunatic can still receive unrestrained, at least the mitigating influences of light and air. As respects the pauper insane, there are few who can be retained with their friends, compared with those to whom an asylum is suitable; but doubtless, in a medical aspect, there are amongst the wealthy patients, who might be advantageously managed in private. In this matter much reflection is necessary. For example, the number and character of the friends by whom an individual will be environed at home, are circumstances worthy of great attention; whether, in other words, they are in the first place persons of intelligence; and secondly, whether there will be such a loving devotion to his care as will eventuate either in his restoration to sanity, or in an amelioration of the morbid symptoms, and which will conduce to secure to him the greatest possible comfort of which he is at all susceptible. The particular features of the patient's disease, and the attendant circumstances generally analogous to those just mentioned, must govern our decision in each separate instance. On this subject, Dr. Jacobi acknowledges his readiness to admit, that many harmless, low-spirited, or hypochondriacal patients, regain their health more easily in the tranquillity of a domestic circle in the country, and under proper direction, than in any other position; the situation itself forming for individual cases of the kind the best remedial means. He afterwards comments on the difficulty of finding persons in private, willing and duly qualified to make the necessary exertions in behalf of those so afflicted. It should not be forgotten also, that a severance from familiar scenes, associations, and persons, is, according to universal experience, almost invariably a measure of advantage in the treatment of insanity.

Most asylums for the insane have not only to be looked upon as curative establishments, but also as adding much to the comfort of a large number of lunatics, who must be considered as decidedly incurable. It is a somewhat different question as to these, and as to individuals labouring under the early and curable stages of insanity. The question now concerns a permanent location: it is, whether a lunatic shall reside as a continuous mode of life in an institution for the insane, or shall spend his days elsewhere? Here the decision to which we ultimately arrive, should also be determined by the character of the patient's mental affection, and his condition as to friends and other modifying circumstances. For if it is evident, that he would be far more comfortable in an asylum than at home, then the abstraction of his liberty in obtaining such a residence is perfectly justifiable. In a medical point of view, perhaps the order of cases causing the most perplexity, are those which fall into a line intermediate between mere eccentricity and positive insanity; these are not usually recent in their origin when first especially observed. What we have to determine is, indeed, whether we shall allow an individual to lead a sort of life most uncomfortable to himself, if we judge his feelings by those of other persons; or shall we bestow on him the comforts of an asylum, whilst at the same time he is averse to a procedure of the kind, has property for his support, inflicts no direct bodily injury on himself or others, and yet lives in a manner which must be painful to himself, or which renders him very annoying to his friends and connexions?

With regard to the second head of the subject that has been allotted to us for discussion, an eminent jurist of Massachusetts remarks, that the right to deprive an insane person of his liberty is found in the great law of humanity, which makes it necessary to confine those whose going at large would be dangerous to themselves or others. And he further observes, that if this were otherwise, we could not even venture to restrain an individual in the delirium of a fever, or in the case of a person seized with a convulsion. Again, as concerns the confinement of those labouring under forms of insanity, which lead them to destructive acts of various sorts, the necessity of this is so apparent, that we may take it for granted that there are regulations to this effect amongst all civilized nations. It is just as necessary to guard the public from being injured by these, as it is to protect them against the violence of real criminals. With regard to interdiction, it may be simply remarked, that under all legal systems, from the Roman jurisprudence down to the different codes of our own time, the grant of this power has been thought requisite. But who shall draw the line of distinction between a form of insanity which is dangerous, and one which is not so; "definitio est periculosa." When we peruse the history of various cases in works on insanity, we find that some of the most horrid acts have been committed by monomaniacs. So also as respects the comparative mental condition of individuals affected with moral insanity; is there any variety of mental disease, which oftener renders its victims unmanageable and exceedingly troublesome? Instances, too, are not uncommon, in which the demented have committed the most fearful outrages. Hence it is that jurists of this country have asserted it to be the *duty* of friends to take the necessary steps for providing a proper degree of restraint to those afflicted with mental derangement; and that in their judgment, although unsanctioned by any statutory provision, their confinement in an asylum is not consequently a violation of a natural right. Hence, also, in an article published during the present year, we find Dr. Winslow declaring that no person evidently deranged in mind, should be permitted to go at large, without some degree of surveillance; and that society must be protected against the insane, and the insane *against themselves*.*

Forsaking, temporarily, the general subject of the confinement of the insane, it seems necessary to touch here on two subordinate points, relative to the same topic. The first of these has reference to that psychological condition entitled a lucid interval. There are many cases of insanity which are periodical in their character; in a ratio with the approach of a patient's mind to complete insanity in these intervals, and with their comparative duration, will he have more or less the right to demand a withdrawal of interdiction and isolation? Each instance, we think, should be determined by its own essential characteristics. And we ought, therefore, to lean to one side or the other; that is,

* *Journal of Psychological Medicine*, Oct. 1850; Art., "The Trial of Pate" (by the Editor).

forbid or allow restriction, in accordance with the degree of the lucidity, its duration, and also the wishes of the individual, and his prospects of self-support when he shall be sole master of his own actions. Difficulties may certainly occur here, but practical good sense should be permitted to disentangle our doubts, and enable us to arrive at a proper conclusion.

A second point of consideration, is the length of time that a patient should remain in an asylum after convalescence has appeared. Now it is manifest that in such a retention we are temporarily confining a man who is sane. This we think, however, entirely justifiable, inasmuch as nearly all writers on insanity agree as to the necessity of occupying due time in the consolidation of a cure; the reports of various institutions for the insane exhibit this fact very plainly. And we should not hesitate in thus restraining a patient, merely to gratify the ultraism of fanatical excitement and visionary theories of liberty. Moreover, we think that a just regard to the safety of the public or of the patient himself, authorizes the retention for a longer period than usual of individuals, who, whilst insane, have committed homicide or attempted self-destruction; for the risk involved in permitting a person to go at large, in whom propensities so dangerous might be still latent, is sufficiently great to require a conviction approaching certainty on the part of a superintendent, that the mental disturbance is removed at the time of discharge.

Having assigned the reasons why a person when insane should be subject to confinement, it remains for us to look somewhat in a contrary direction, by turning the view to abuses which have attended the exercise of this power of isolation. Individuals merely eccentric, or altogether unaffected in mind, have been incarcerated, not for their own good nor for the safety of the public, but only with the pretence of insanity to carry out evil designs on their property or to serve some other unholy purpose. This has been an occasional result in many foreign countries, though we have scarcely heard of any cases of the kind in the United States. As concerns American asylums, the very few supposed examples, in which a portion of the public have deemed otherwise, in our opinion were wholly fallacious.

There are, then, two purposes to aim at, in instituting legal provisions for the confinement of the insane. First, that the advantages inuring to hospital treatment, and particularly as regards recent cases, should be fostered, as far as possible, by a wise legislation. And secondly, that all abuses should be subject to correction, by the invariable establishment of a watchful and entirely paramount supervision—a supervision by its characteristic features removed to as great an extent as is feasible in human affairs, from the probable action of selfish motives. With regard to the first of these principles, circumstances must so vary its action, that we have no space to enter into details. For example, the means of support possessed by an asylum, or by the patients therein, the extent of a country, the reputation of an institution, and other modifying influences. On the whole, it may be remarked, that whilst the laws, in appointing an authority to judge of a person's sanity and take the responsibility of confining him, should be such as will ensure the deliberate action of an unbiassed tribunal, at the same time they should never be so complicated or of a nature that would create difficulties in sending an insane patient to an hospital, at an early stage of his mental disease. Perhaps some legislation is needed here in most communities; for it is a common cause of complaint with medical superintendents, that the insane are but too often kept back from asylums until they have become wholly incurable. Respecting the prevention of false imprisonment, the great measure for this end is embodied in an aphorism of Millingen, to the effect that "All lunatic asylums, whether public or private, should be placed under the immediate care of government." With regard to the steps made necessary for restrictions on the insane elsewhere than in an asylum, a carefully devised local tribunal is not an entirely sufficient safeguard against corruption; there should also be some central authority emanating from the government of a State, and having wide powers of investigation.

We cannot avoid thinking, moreover, that the free entrance of visitors into asylums has an excellent tendency in relation to their custodial functions. Under this regulation, in instances in which the reputation of an institution is jeopardied, pre-conceived notions on the part of communities, or an entire want of previous reflection, are not so liable to exert a pernicious effect. If, for example, the accusation is raised that sane persons are confined in the demesnes of a hospital, there will be most probably a number of visitors who can contradict such a report. We waive here the question as to the moral influence upon the insane from the introduction of visitors, but would simply remark, that our opinions on this point have been heretofore expressed, and that our views and experience are directly opposite to those of most of our medical brethren.

In conclusion, we may venture to observe, that although as to the confinement of persons in asylums, public opinion is often in the wrong, yet caution is requisite from the managers of these charities, lest an institution should be placed here in a false position. If the opinion and action of the public are evinced decidedly against the confinement of an individual as being an unfit subject for the process, although to those experienced in the symptoms of insanity the reverse may seem clear; admitting also, that the legal right of restriction is fully in the hands of those having charge of an asylum, it is still, to say the least, doubtful whether this right should be exercised. The matter evidently stands thus: so far as the welfare of the institution is concerned, it makes no difference whether the supposed lunatic is committed to its care or not; but by insisting on the use of a lawful power, it inevitably gives colour to the accusation of improper motives. In our judgment it would be better to yield to the wishes of the community, at the same time making a public protest against the erroneous train of ideas by which they are deceived. This we believe, too, is the proper course, not only to avoid the false imputation to which we have referred, but for the especial reason in addition, that in a republic, respect is always due to the opinions of the people.

